# A qualitative study of clinical narrative competence of medical personnel

**DOI:** 10.1186/s12909-020-02336-6

**Published:** 2020-11-10

**Authors:** Shao-Yin Chu, Chin-Chen Wen, Chi-Wei Lin

**Affiliations:** 1 Department of Pediatrics; Department of Medical Education, Buddhist Tzu Chi General Hospital, Hualien, Taiwan; 2 Department of Medicine, College of Medicine, Tzu Chi University, Hualien, Taiwan; 3 Department of Human Development and Psychology, Tzu Chi University, Hualien, Taiwan; 4 Department of Counseling & Clinical Psychology, National Dong Hwa University, Hualien, 97401 Taiwan, Republic of China

## Abstract

**Background:**

Medicine practiced with narrative competence is called narrative medicine, which has been proposed and used as a model of humane and effective medical practice. Despite the in-depth discussions of narrative medicine, the study of narrative competence in literature is limited; therefore, this study aims to explore the dimensions and connotations of the clinical narrative competence of medical personnel.

**Methods:**

This qualitative study used in-depth interviews to collect participants’ experience and perspectives regarding narrative competence, followed by thematic analysis of the transcripts**.** Through purposive sampling, this study successfully recruited 15 participants (nine males and six females in 2018–2019) who were engaged in narrative medicine or medical humanity education from different medical schools and hospitals across Taiwan. The authors performed manual thematic analysis to identify the themes and concepts of narrative competence through a six-step theme generation process.

**Results:**

There were four major themes of narrative competence generalized and conceptualized: narrative horizon, narrative construction (including narrative listening, narrative understanding, narrative thinking, and narrative representation), medical relationship (including empathy, communication, affiliation, and inter-subjectivity), and narrative medical care (including responsive care, balanced act, and medical reflection). These four themes were further integrated into a conceptual framework and presented in a diagram.

**Conclusions:**

Cultivating narrative competence in medical education can complement traditional biomedical orientation. Regardless of their treatment orientation, narrative medicine-informed health practitioners may take advantage of their multi-dimensional narrative competence, as presented in this article, to enhance their awareness and preparation in different areas of competence in medical services. In addition, the results of this study can be used as a framework for the development of the behavioral indicators of narrative competence, which can be taken as the basis for medical education curriculum design.

**Supplementary Information:**

The online version contains supplementary material available at 10.1186/s12909-020-02336-6.

## Background

Medicine practiced with narrative competence is defined as narrative medicine, which has been proposed and applied as a model for humane and effective medical practice [1]. The ability to acknowledge, absorb, interpret, and act on the stories and plights of others are the essential components of narrative competence. Narrative medicine is an innovative model for improving health outcomes, and along with scientific ability, physicians need the ability to listen to the narratives of their patients [1].

Due to the influence of different schools of thought, including medical humanities, cultural studies, literary theory, social sciences, feminism, psychoanalysis, the work of Michael Balint, primary and patient-centered care, biopsychosocial medicine, holistic care, and postmodern ideas [2–7], the concept of narrative medicine emerged late in the twenty century in response to the inadequacies of, but not to dispense with, the biomedical model [6–8]. After teaching a multidisciplinary course to general practitioners, practice nurses, health visitors, and other primary care professionals since 1995, Launer found that this narrative approach can be useful for not only mental health problems, but all medical encounters [7].

Studies of primary care generally show a strong correlation between quality relationships and patient satisfaction. In addition to medical skills, other physician qualities, such as listening, interpersonal skills, empathy, and commitment, are highly valued by patients [9]. As Roberts stated, the trend (so-called post-Balint) in medical services moving to more interpretive and empathic medical consultations is not just a shift to more psychosocial models, it is also a shift in interactional models. In accordance with Balint’s work, Charon pointed out that narrative medicine provides the means to understand the personal connections between patient and physician, while simultaneously offering physicians the means to improve their effectiveness to work with patients, themselves, and others. Practicing with narrative competence enables physicians to form empathic engagements with patients and establish therapeutic alliances [10]. On the other hand, narrative medicine or narrative-based medicine enables patients to unburden themselves, and attentive listening is intrinsically therapeutic [6], while “the narrative provides meaning, context, and perspective for the patient’s predicament” [11].

Western medicine in the post-1990 period presented an important trend of reflection. It was thought that the traditional medical treatment presented a method of reductionism. When collecting and understanding a patient’s complaint, physicians used a logic-scientific method to analyze, classify, and develop a reasonable diagnosis and treatment strategy. However, in order to provide holistic medical care, it is necessary to take into account the psychosocial aspects, in addition to the disease, the family and social cultural context, the patient’s spiritual beliefs, and the other impacts of medical care decisions. As the role of digital technology in clinical medicine becomes more important, less attention was paid to the personal narrative from the patient’s subjective experience [12]. Therefore, medical personnel need to have appropriate “narrative competence” as well as medical-related knowledge. In addition to making correct diagnoses for patients, medical personnel need to understand the relevant background stories of the parties and be able to describe and explain their psychosocial intentions in order to develop a medical plan from the perspective of the patient. This trend of reflection emphasizes the importance of narrative thinking and has had a resounding effect in the field of medical education.

Charon suggested that physicians read literature to promote narrative thinking, and engage in reflective writing regarding the stories of patients, themselves, colleagues, and society, in order to enhance respectful, empathic, and nurturing medical care in narrative medicine and medical education [1]. When learners wrote and reflected on the events that had impact on them during their provision of medical care, they may describe and elucidate something not taught in school, find their professional identity from a good role model, and experience the art of empathy through patient-professional encounters [13]. The modes of reflection include taking a break from a difficult practice or action, reviewing this experience, and reassessing to find problem solving methods based on the reality and multiple possibilities [14].

The human experience has a storied nature [15]. Humans organize life experiences through narratives [16]; through self-defining life stories, they understand their lives, and provide a kind of identity, meaning, and coherence of individual lives by the self-narrative methods of reconstructing the past and expecting the future [17]. That is to say, the emphasis on the narrative nature of individual psychology and the discussion of human experience through narratives provide an inquiry orientation that is different from traditional positivism in the exploration of human life experience [18–20].

Narratives can be seen everywhere in medicine. For example, patients in the clinic and in the wards tell the doctor about their conditions; the physician listens to the patients’ narrations and further integrates the information heard into the medical knowledge to explain the cause and treatment to the patient. Outside the clinic and the ward, physicians communicate frequently with colleagues in different professions such as dietitians, social workers, and especially nurses. In addition to the narrative behavior of inter-disciplinary cooperation and mutual trust, these communications rely on the re-narration of the patient’s disease story to provide a whole-person medical care that meets the patient’s needs. More importantly, medical personnel with narrative competence can not only understand their emotional reactions to patients but also actively enter the patient’s story, listen to and communicate with each other’s concepts, and construct and implement mutually understandable health care behaviors and cultures.

Shared understanding is created through conversations, meaning that doctors and health professionals can allow patients to adequately express their stories in their own words, explore connections, differences, new choices, and possibilities, and deliberately probe and guide the dialogue to promote understanding without being controlling, interfering, or indifferent [6, 7, 21]. Exploratory questions, such as “What does this mean for you?” and questions and prompts that invite change, such as “What needs to happen for the situation to change?” can be used to facilitate communication and treatment. By being curious, doctors and other medical personnel, can listen to patients’ narratives [2] and pay attention to the contexts and complexities of the medical encounters, as well as the circularity nature of the interactions or influences involved, in order to provide nonjudgmental and genuine care that is sensitive to patients’ needs and displays a readiness for change [6, 7].

What kind of narrative competence is demonstrated by the experience with such narrative nature? This can be seen from the two perspectives of narrative knowing and thinking. Generally speaking, formal science is more about category knowledge. It defines categories by operational definitions and understands the relationship between categories through attribute relationships. Narrative knowing is a form of constructing knowledge based on the schematic format. According to Mandler [22], schematically organized knowledge may constitute: 1) spatial schema, which is the schema of the “scene”, to understand from the partial-total spatial relationship, and to think that part is not an independent part, but a part of the whole space; 2) temporal understanding, which is the schema of the “plot” and the understanding adopted by many story narratives, such as biographies. Based on this perspective, narrative knowing means that concepts or ideas are organized into systematic relationships through a whole or theme. From the perspective of narrative thinking, when people face a stimulus, they do not directly respond to it. Instead, they absorb and compare the stimulus with the existing psychological patterns and engage in various cognitive activities to form meaningful messages. This is an assimilation process. When people face textual stimuli, they are assimilated into the internal representation of the narrative grammar that has been acquired [19]. Moreover, narrative thinking involves how individuals understand the actions of others and how individuals are related to others, because the weaving of a story is a collaborative activity that explains why the narrative produces mutual understanding [23].

Rita Charon, a pioneer of narrative medicine, has long been committed to the promotion of narrative medicine and the training of practical work. Charon believes that narrative medicine is a narrative science with a theoretical basis that includes: narrative theory, biographical theory, phenomenology, theory of mind, traumatic research, and aesthetics [24]. Inspired by the field of literature and linguistics, she believes that the narrative competence is the acknowledgement, absorption, and interpretation of the human disease story and being moved to action by it, which can be applied to the clinical work of medical care [1, 24]. However, in different literature, narrative competence has various connotations, such as practitioners with narrative competence showing empathy, reflection, professionalism, trustworthiness and other characteristics in clinical circumstances [1], or narrative competence as a combination of textual skills, creative skills, and affective skills [2]. Textual skills involve the confirmation of story structure, the adoption of multiple perspectives, and the understanding of metaphors; creative skills involve a variety of interpretations of imagination, curiosity, or the creation of multiple endings, etc.; affective skills refer to the tolerance of the uncertainty of the story or the emotions of entering the story, etc. In practice, narrative medicine emphasizes three elements, namely: attention, representation, and affiliation [25]. Attention means not judging and criticizing, being able to have an open mind to listen to patients, and being able to receive messages from patients and their families. Representation is the further reflection and integration of what is heard and then reorganizing and interpreting the message by means of words or images to summon the testimony and hope. Affiliation is a combination of attention and representation, which can form close relationships, cross the gap between doctors and patients, and connect with patients through narratives to create links and promote medical relationships. Charon’s view of narrative medicine is a perspective closer to literature, aesthetics, and philosophy. However, how to turn these connotations into specific measurable narrative competence indicators needs to be further explored.

This paper suggests that the evaluation of the effectiveness of narrative medicine education and training is not only based on the change of attitudes or perceptions of learners/participants but also the display and enhancement of narrative competence. Therefore, the main purpose of this study was to explore the conceptual connotations and items of the narrative competence of medical personnel from the perspective of narrative medicine in order to provide a reference for the improvement of the quality of narrative medicine education in the future.

## Method

This qualitative study used an in-depth interview method to collect the experiences and perspectives of the narrative competences of Taiwanese medical personnel engaged in narrative medicine (including medical humanities), followed by thematic analysis of the transcripts. The purposive sampling method was adopted for the interviewees. There were 15 research participants, including nine males and six females. Of the participants, nine were doctors and nursing staff responsible for medical education and six were general or humanities teachers in medical education (see Table 1).

**Table 1:** The demographics and specialty of participants

Participants’ code	Gender	Degree (Major)	Specialty of medical education
LJ	Female	PhD (Medical anthropology)	Medical humanity, Medical history
SZ	Female	PhD (nursing)	Narrative medicine, Medical education, Mind-fullness practice
YW	Male	MD (Family physician), PhD (Public Health)	Medical humanity and ethics, public health
YH	Female	PhD (English literature)	Medical literature, Narrative medicine
WD	Male	MD (Anesthesiologist)	Medical literature, Narrative medicine
DJ	Male	MD, PhD (Anthropology)	Medical humanity, Cultural and historical sociology
CW	Male	MD (Neurologist)	Medical humanity and ethics, Medical accreditation, Medical writer
MH	Female	PhD (Linguistic)	Medical humanity and ethics, Linguistic analysis
CH	Male	MD (Neonatologist)	Medical education, medical accreditation,
JD	Male	MD (Thoracic physician)	Narrative medicine, Medical education
XY	Male	PhD (Philosophy)	Narrative medicine, Professionalism, Life ethics
HR	Female	PhD (Philosophy)	Medical ethic, Philosophy, Phenomenology, Ethics
FZ	Female	PhD (Nursing)	Medical humanity, Medical anthropology
SJ	Male	MD (Pediatrician)	Narrative medicine, Medical anthropology
ZG	Male	MD (Family physician), MS (Anthropology)	Narrative medicine, Medical anthropology, Illness narrative

The interview protocol (interview guide) used in this study was developed for this research (see Additional file 1 for the online version). The authors initially drafted an interview protocol, as based on the research purpose and literature review in the study plan. After obtaining approval from the institutional review board at the Baddish Tzu Chi General Hospital (IRB106–141-B), we engaged in a pilot interview with a medical teacher, and then, revised the interview protocol according to question-answer clarification and feedback.

The interviews were conducted by the authors of this study. Each interview began with an explanation of the purpose of the study and an invitation for the respondent to fill in the interviewee’s consent form, followed by a semi-structured in-depth interviewing. The order of the questions was not fixed until all questions from the interview outlines were covered. The interview outlines included:

1. Please introduce your medical (or professional) background, training and practices.
2. Under what circumstances or occasions were you exposed to narrative medicine (NM)? What are your relevant training, education, and research experiences regarding NM?
3. What is your viewpoint and concept of NM? Are there any similarities or differences between the current mainstream logic and scientific-based medical knowledge?
4. What is your understanding and opinion about the concept of narrative competence (NC) of medical personnel?
  4-1. What is your understanding and opinion about Rita Charon’s definition of NM and NC? (We provided the definition of NM and NC by Rita Charon before the interviews.)
  4-2. What are your understandings and opinions on the definition of NM and NC by other scholars?
5. How do you incorporate the concept of narrative medicine into your teaching activity?
6. How do you incorporate the concept of NM into your practice?
7. What do you expect the differences in the students’ clinical practice will be after receiving narrative medicine training?
8. What is the difference in your care for patients after incorporating narrative medicine in your clinical practice?
9. What will be the obstacles (questioning, challenges, and resistance) to promoting NM in medical education and clinical practice?
10. What narrative competencies are necessary for medical students in your medical education experience?
11. What narrative abilities are needed for medical personnel in your clinical experience?
12. Which narrative competencies are necessary for medical students in your medical education experience?
13. Which narrative competencies are needed for medical personnel in your clinical experience?
14. What narrative competencies do you think can be applied, and how can they be applied in clinical care?
15. Others

Before data analysis, the interview recordings were transcribed into text, all identities were hidden, and any unclear parts were repeatedly listened to and clarified. Furthermore, thematic analysis was manually conducted by four coders (three authors and an external professional colleague), who independently reviewed the materials and jointly conducted a six-step theme generation process [26]. In Phase 1, repeated reading was conducted to gain an overall understanding of the text. In Phase 2, the initial codes were generated from the entire data set for each individual interviewee. In Phase 3, the initial codes were collated into themes, where the main overarching themes and subthemes were identified. In Phase 4, the validity of the initial themes and subthemes in relation to the data set were reviewed. In Phase 5, the themes and subthemes were further defined, refined, and named. In Phase 6, vivid examples were chosen for each theme, and the relations between themes were conceptualized into a diagram.

The credibility of this study was established through triangulation [27, 28]. Specifically, this study took participants from a wide range of educational and clinical backgrounds, including medical humanities and different medical specialties, to achieve “source triangulation”. In addition, in order to establish an “analyst triangulation” that requires external reviewers or multiple analysts, our team (the three authors, and a professional colleague who served as an external co-analyst) worked together to analyze the transcripts until consensus was reached, as this analysis process helps to facilitate discussion and clarify possible blind spots.

## Results

The main results of this study are shown in Table 2. The concept connotations of narrative competence included four main themes and 12 subthemes.

**Table 2:** Concept Connotations of Narrative Competence

Theme	Subtheme	Concept
**Narrative horizon**	• Narrative horizon	Patient centered; peace; authentic effect; healing
**Narrative construction**	• Narrative listening	Attention & interesting; key words; nonverbal messages; observations; humanistic sensitivity
	• Narrative understanding	Patient perspectives; life history & critical events; psycho-social-cultural context; sufferings; strengths
	• Narrative thinking	Illness temporality; authenticity; meaning making; narrative inquiry
	• Narrative representation	Phenomenological articulation; decoding & interpretation; metaphor & creativity; life related to illness
**Medical relationship**	• Empathy	Listening; perspective taking; being moved; emotional sensitivity; connecting to patient
	• Communication	Facilitating; empowering; responsive; intra & inter-professional communication; sharing life experiences
	• Affiliation	Mutual empowerment; rapport; sharing burdens; acceptance & appreciation
	• Inter-subjectivity	Constructions from medical personnel & patient interactions; ethical thinking; inter-professional collaboration
**Medical care**	• Responsive care	Patient-centered care; responsive to patient’s needs
	• Balanced acts	Sharing decision making; bio-psycho-social-cultural considerations
	• Medical reflection	Self-awareness; tolerance for uncertainty; self-reflection/in action/for action/of action

### Narrative horizon

The narrative horizon/perspective refers to the narrative medicine views and beliefs held by medical personnel. The narrative perspective is patient-centered, and places emphasis on individualized interventions and patient subjective experiences: meaning medical intervention is not just the elimination or relief of symptoms, but rather the further pursuit of peace and wellness, that is, caring for the patient’s physical and mental care needs, as well as personal and family adjustments related to such medical care. Patient-centered care is:

*“When the patient is taken as the center, there must be an illness history. The illness history is a process of the patient’s illness. This is the beginning of the narrative. You have to understand his entire process. (YW-007) The illness history includes the parts of his stress, his family, his profession. (YW-009) Narrative medicine is also training us to understand a patient’s more complete story, not just some situations, such as when he had a fever, when he had a headache, what was the situation when he had a fever or why did that happen at that time?” (YW-019).*

In the perspective of narrative medical care, medical professionals do not limit medical treatment to just the facts or events that have occurred; they see patients and peers as teachers, and examine the course of treatment, the medical relationship, and the patient stories to find moving emotion and inspiration. Interviewee SJ mentioned a sense of authenticity:

*“The authenticity of our lives is a kind of sense of reality, not truth. Truth is cruel, unacceptable, and there is no way to change it, [ …] if you go to see a person who has been injured from a fall at a very young age, and has paralysis of half the body, what should he do? How would he spend his life? It is hopeless for many people, but, if we have narrative medicine, we have many ways to help him change his...” (SJ-025).*

Finally, medical care works do not end at a cure, but extend to healing, including empathy, support, and responsive care; moreover, it assists clients in perceiving, understanding, accepting, discovering meaning, and enhancing self-motivation. Interviewee SJ mentioned a case where even patients with end-stage cancer can experience peace of mind:

*“There is a patient who also has the kind of hydrovarium ovarian cancer. The hydrovarium is already full, [ …] Her eyes are very determined, and she uses a brush to write, and then, draws the Guanyin [a religious fugure] image. She painted it very well, [ …] She can still move like this when she is dying, [ …] She said that she is breathless, but can remain calm, so spirituality is still very important! Isn’t it? That is the ultimate goal of our narrative medicine, the hope to be able to achieve spiritual peace.” (SJ-023).*

### Narrative construction

Narrative construction refers to medical care, as based on the spirit of the narratives. The concept of this theme is rich in content and can be divided into four subthemes, namely:

#### Narrative listening

The narrative spirit is constructed with emphasis on “the interest in the patient and attention to the person”, meaning being able to listen to the key words or implications of the patient’s words, while simultaneously listening to the patient’s nonverbal narrative, and being adept at observation. As an interviewee mentioned, *“There are some doctors [ …] If he had not cut off the interview so quickly, if he had given a little more time for the patient to talk, the narrative would have appeared sooner.” (MH-006)* It is important to have humanistic sensitivity, that is, to be able to perceive the patient’s feelings and thoughts, notice the patient’s key narratives (including differential narratives and special narratives), discover the relevance of the disease to the individual, learn about their localized life experience related to the disease, and be able to remain sensitive under unreasonable circumstances. For example, interviewee HR believes that, whether medical staff *“have interest in patients or not” (HR-037) is very important, and narrative competence is nothing more than a skill.”*

*“You have to be sensitive first. Sensitivity is to read, listen, and feel, and there should be training for it, so that you can see more and deeper than others…, and then think more and in more detail, and listen more… We need to be trained for this kind of sensitivity, actually this is the sensitivity of the humanities… The so-called sensitivity is to be able to identify, like Rita Charon, it is to acknowledge, and to acknowledge means that you want to pick up those things, those implications.” (CH- 019).*

Narrative listening is like having a literary soul: *“You can absorb his story, and then you can understand, and then you can be touched, and then you can actually pay attention! Did the first patient come in and feel that he caught your attention, that is…did he think he was noticed by you! That, that is more than the first… the first moment. He is also judging whether you, as a doctor is… caring” (HR- 053).*

#### Narrative understanding

Narrative understanding focuses on the patient’s perception and interpretation of the disease, that is, “*the patient’s model of interpreting his disease, how does he think he is sick? What kind of disease does he think he has? Then how long before he feels he will be better? [ …] What does he think of the disease? Or how does he feel?”(LJ-029)*; and *“What kind of network does the disease occur in, and what kind of patient world does this network construct for the patient? …*” *(FZ-007)*, thus, understanding the key events of the patient’s life history and their relevance to the disease, and especially, inter-generational understanding, are important.

*“It may be difficult for young nursing staff to understand the course of the past life of veterans. There were so many critical events in their lives. In fact, the critical events are when he was fighting or when he was away from home. The point is, in fact, when we are taking care of him, at the end of his life, when he is dying, or in the process of his hospitalization, and those moments will actually cause him to be in a certain state of mind. So, there are a lot of critical events for patients that are actually very important.” (DW-008).*

Rather than focusing on physiological conditions, narrative understanding focuses on the psychological, social, and cultural context of the disease, interviewee MH said:

*“You need to use biophysical orientation to connect to the psychosocial, [ …] Suppose the patient just now, the patient who got the headache, [ …] I asked how long this headache has been hurting, and then asked, do you think there is a reason that caused your headache today [ …] He might say how much pressure the company has given him, [ …] This is what we are coding, when it comes to this, it means when you see he has a headache, you should proceed like this, then generally, you will get such psychosocial information.” (MH-020).*

#### Narrative thinking

Narrative thinking is not meant to arrange events in chronological order, but to place the disease/hospitalization in the context of life to construct the temporality of the disease.

*“A person’s life is lived in decades. In fact, his hospitalization took place only at a certain point of time, then how do you connect this point to his past and his future. His hospitalization, our hospitalization preparation plan, his life in the future, and his life in the past, how do you connect all these in series…” (DW-086).*

In addition to biomedicine orientation seeking the truth and facts, narrative thinking focuses on the authenticity of patients and diseases, which refers to touching stories and revelations in medical care, including the value of life, the beauty of human nature, selfless dedication, resistance to disease, family emotions, loss, rebirth, etc. As Interviewee FZ stated, *“You see this patient as glorious, or the kind of attitude when a person is facing life ... maybe it is also a kind of wrestling match to some extent, there are some people who wrote articles like the Old Man and the Sea.” (FZ-060).*

The traditional biomedical orientation focuses on the provision of information or lab tests, while narrative medical orientation emphasizes creating meaning, including concatenation and interpretation. Narrative medicine emphasizes narrative inquiry, including open-ended questions and exploratory findings, other than regular questions.

#### Narrative representation

Narrative representation focuses on phenomenological description, which can specifically describe the details, while creating a narrative reproduction that is close to the phenomenon. Interviewee SJ used an example to illustrate:

*“Just like we see diabetic feet, we dare not describe them, that is, narrative medicine emphasizes that you are going to face that wound, and then go on to describe the appearance, not just to name it indirectly as a bad bacterium, like this is one, this is just some kind of bacterium. It is not like this. Instead, you should tell what a wound looks like, describe it in detail, and then, let everyone discuss it...” (SJ-017).*

Narrative representation comprises interpretation and decoding by medical personnel, as well as making good use of metaphors and the imagination to connect the disease with the life aspect. Imagination is not illusion; instead, it is a method *“ … that can be used to make a more reasonable statement of this entire event. It doesn’t necessarily fully conform to the facts, but they can collide, and it can really test the imagination. [ …] On the other hand, a narrative can actually give him a little more guidance, to view this matter in either a plane or a 3D stereoscopic way. “ (XY-055).*

Interviewee SJ further illustrated the competence of representation:

*“For representation, some people will think that it seems to be just to say it! Actually, there are a lot of skills in it. Those skills include your life experience. If you can connect a lot of things happening to the patient with your life experience, and then, use another more specific thing to explain this thing that is happening now, it often results in a kind of great, great shock...” (SJ-045).*

SJ went on to explain the use of the metaphor:

*“For example, cancer, how that kind of cell transfers, just like our most commonly used metaphor is war [ …]*, *you are a warrior! You are a soldier! (SJ-046) “We are some supporting characters in your scene, and you are the protagonist [ …] When tumor metastasis happens, it is like enemies are setting fires everywhere, how do you contain the fire, and quickly transfer troops, what submarine do you use, what do you want, that is, how to convert all kinds of healing weapons into various weapons, how to attack, [ …] You can use various war metaphors, yes.” (SJ-048).*

### Medical relationship

The medical relationship based on narrative medicine includes three subthemes:

#### Empathy

A narrative-oriented medical relationship is based on empathic listening. Medical professionals must put themselves in the position of the patient, treat patients as family, be able to judge another person’s feelings by their own, have the rapport ability to be emotionally moved, and be able to establish a link with the patient. However, empathy and sympathy are different. *“Sometimes we all mistakenly think that it is sympathy [ …] In fact, most patients do not want sympathy, [ …] Why should you sympathize with me, I have lung cancer, not you … You come to see me now, why is it that you want to come and see me, some of them will be negative...” (JD-126).*

It is important to be able to judge the other person’s feelings by one’s own: *“Every one of us is a book. You can understand the logic of that book only with the narrative, and with the narrative to understand the logic of that book, you will find that you have the emotional link of judging the other person’s feelings by one’s own, and you can understand everyone’s unique logical reasoning... ”(DJ-138).*

#### Communication

The core of the medical relationship is communication, and medical personnel should be able to facilitate patient narratives and have the skills to simultaneously empower them to respond and provide feedback appropriately to patients. Interviewee MH mentioned an elderly case:

*“If you think about him in his 80s, and he comes alone for a hospital visit [ …] If this is a first-time visit, this situation is really rare, so you are a little sensitive, and say, “Oh, you came here alone today”, then some people may cry soon after hearing this, while some people may talk […] “the children do not have the time”. Then you will say, “Oh, you are great, you can take care of yourself”. Look, if you say one or two sentences at the beginning like this, that relationship will become very different...” (MH-016).*

Medical relationships include communication between doctors and patients, as well as across medical professions. The exchange of life experiences and dialogue between doctors and patients is one of the steps in the promotion of good medical care.

*“When the next treatment step is made, his views will be considered. Then, the establishment of such communication relationship between patient and the doctor must be more than saying how many times he takes the medicine per day or asking if he is smoking. Then, he will tell the truth, because if he continues to smoke, you have to understand what his environment is, and when it started. He may be smoking because everyone in his work environment is smoking...” (CH-008).*

#### Affiliation

Affiliation in the medical relationship means that doctors and patients can be mutually empowered, establish a good congenial relationship, share the burden of the disease, and have the ability of feeling affection, in order to find acceptance, be grateful, etc. Interviewee SJ further explained the meaning of affiliation, as follows:

*“Affiliation is to achieve a kind of tacit understanding with the family, that is, understand the experience by sitting down together and sharing together [ …] Two people bearing a burden is better than one, because if he accepts you, he will be more willing to allow you to share his burden, and his burden will be lighter, just like a best friend [ …] When you reach such affiliation with the patient, that thing can be put aside, you don’t have to worry about what kind of thing it is, so that allows the patient to reach a peace, at least temporarily...” (SJ-027).*

#### Inter-subjectivity

Compared with the construction of expert subjects centered on medical personnel, the construction of narratives emphasizes interaction with patients, promotes inter-subjectivity, and constructs meanings from impressions into contexts.

*“Subjectivity is constructed with each other. If there is a patient constructing with a doctor throughout this entire process, I think the career of this doctor will be pretty good. (XY-014) Narrative medicine can make the doctor himself a reader. It is like reading to the patient, [ …] like when we describe the story, it is the impression points, and these impression points are connected in series to become a narrative, then, how does he interpret this hospital as treating his patients, how does he interpret how his entire life processes are treated, including interns and nursing staff, [ …] he is constructing, so he actually treats himself as a text, to a certain extent, if his text is in the direction of cooperating with you for construction, I think this is a positive direction.” (XY-064).*

Ethical thinking and action are important parts of narrative subjectivity, including being able to understand others and transcending differences, being able to understand and reflect on values, making trade-offs and balances between different values, and having cross-disciplinary cooperation with different professions in the end. It is important *“not to let the subjectivity of different objects be erased by you.” (HR-012).*

### Narrative medical care

Narrative-centered medical care has three core subthemes:

#### Responsive care

The medical treatment of narrative medicine is individualized patient-centered care, as based on working with patients to build a trusted medical relationship and developing care that responds to patient needs, and this kind of care is different from the reductionist attitude:

*“The reductionist’s attitude is to be able to see how many patients are seen during the morning. This is how reductionism is related to this. But, to teach students, what we want is individualism, we want to make their patients different from ordinary patients. The most important thing is if you can see what the patient’s need is.” (CW-073).*

Medical personnel must actively listen to the issues of concern from patients, invite patients to join the medical team and become team members, and give patients the opportunity to express what they want and the medical model they are looking for. Most importantly, patients should have the opportunity to participate in medical decision-making together, in order that the patients’ real needs are considered.

#### Balanced act

Narrative-oriented care not only focuses on physiological issues, such as characterization of the disease, new diagnostic techniques, empirical treatment outcomes, side effects, and complications, it also considers psychological and emotional symptoms, such as patient anxiety and worry, fear and the expectation of medical care, behaviors adapted to the disease and treatment, and related issues of family, cultural system architectures, and social contexts. Interviewee LJ told a related case story that illustrates the importance of seeing the patient’s feelings during clinical care:

*“The patient is conscious of his appearance; though he wears patient clothes, he puts on different scarves every day [ …] Why would he resist the bladder training [ …] the urine bag is so obvious, will the urine bag smell bad? Will there be obstacles to his wish to look nice [ …] So, in the process, they thought a lot about how to communicate with him, and finally, the patient was willing to accept it, and then, even very grateful, because they helped him, knowing that he is still keen on looking nice, they made his urine bag smaller, meaning he could neatly put in his pants, and the patient was so happy!” (LJ-035).*

Medical professionals take into account the psychosocial issues that affect the treatment of diseases, such as the impact of the disease on individuals and families, the role changes, financial problems, how patients are treated by relatives and friends, the nature of their work, and even spiritual issues, such as faith and spiritual support, which are parts of balanced medical acts.

*“Because like us, whether as physicians, nurses, or medical staff, we all have a clinical goal, and that is to assist this patient in this care, [ …] For example, they want to emphasize the body and mind, but our clinical part is only about the body. While the psychological part is also there, it may just be we have not asked about it (LJ-014) or we may not be able to handle it. At most I ask the psychologist, I look for social workers, or I talk to him, [ …] When I am about to enter the ward, I don’t know what story I will encounter, but then, I would slowly teach them how to identify the stories related to their care...” (LJ- 015).*

Furthermore, healthcare providers must pay attention to the balance of personal life, mind, and body, and seek reconciliation and responses in their various medical works and personal life factors (including resistance, hesitation, and challenges). Maintaining appropriate medical relationships, while avoiding burn out, is also a practical and important balancing method.

#### Medical reflection

Narrative medicine focuses on medical reflection and includes: recognizing and tolerating medical uncertainties and individualized medical needs; the self-reflection and self-consciousness of medical personnel (who can feel a sense of their own feelings and can self-monitor their physical and mental state); reflection in action (such as, how to effectively complete medical tasks, how to use evidence-based medical care, and how to pay attention to and evaluate medical quality); reflection after action (such as, how to identify a problem, identify a root cause, or make future improvement plans); and reflection on future actions (such as, how to continue self-directed learning and progress and reflect on the nature of life). Interviewee SJ mentioned a good reflection metaphor:

*“It’s like a trout in the rapid current. She suddenly finds some stones to hide behind and stays there, because the vortex there is not that big, and then, there is the purest oxygen, aren’t we like this? There are not so many hours of sobriety, they are taken away by others, we are just looking for them, and we suggest (medical) to students every day that they need to find time to think about what happened today, [ …] that is, you can review in this short times of three or five minutes, what happened to you today, what enlightenment you got...” (SJ-025).*

Interviewee FZ also offered a good explanation for inspiration from a patient’s disease story:

*“Understand the patient as a book, and then, when you are reading this book, you can actually understand him from many angles, or you can actually have a lot of life conversations with him, including citing from your own life experience. In fact, there may be some kind of communication or exchange between you and a patient, and this will let you know about life or death and disease in your clinical work [ …] He has a certain experience of life, and in fact, he is standing in one of the richest fields, and then, an observation and introspection offered in this area can help him continue to go on, and this is a very good way for doctors to practice medicine.” (FZ-007).*

**Fig. 1:**
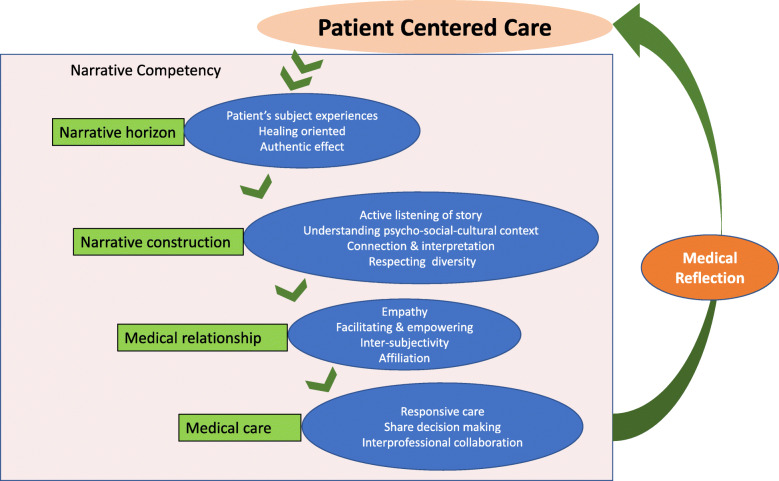
Integrated diagram of narrative competence conceptual framework

This study further integrated the above concept connotations, and formed descriptions and a diagram of the conceptual framework (see Fig. 1). When a patient enters the medical system, guided by the patient-centered core horizon, medical personnel can pay attention to the narrative framework of the patient and the authenticity of the disease, and begin to form a narrative construction. First, they must actively listen to the patient’s disease story, and carefully observe and understand the physiological, psychological, social, and cultural factors in the patient’s illness story, as well as the key events in their life course. Furthermore, medical personnel can use narrative thinking, pay attention to the touching stories to achieve enlightenment for medical treatment, link the disease to life through such inquiry, and find connections and interpretations of the narratives. Furthermore, through such inquiries, they can reach across differences, treat the patients as family members with empathy, and establish a medical relationship of inter-subjectivity and affiliation through the communication skills of facilitation and empowerment. In clinical care, medical personnel should provide patients with responsive care and patients should participate in shared medical decision-making. Moreover, medical personnel must be able to engage in self-reflection from time to time throughout this process.

## Discussion

The results of this research were constructed based on the experience and practice of medical education experts and scholars. The research results could be important for the development of narrative-oriented medical education.

Narrative based medical knowledge, starts from the patient’s point of view and invites the patient to describe incidents that he or she cares about, which are not necessarily related to the disease itself but could be about the illness, the impact of the disease, or the patient’s perception and emotion of the disease. These are all subjective expressions and are associated with the social and cultural background, personality, growth history of the patient, and values. Patients often use metaphors or symbols to explain their conditions. Each patient has diverse intentions and issues that are non-generalizable, and each patient has a unique narrative. Physicians and medical personnel with narrative competence can appreciate the patients’ illness stories and their fight against disease, and they will be more consciously able to help their patients reshape their life stories through medical relationships and patient-centered medical interventions. Under the efforts of cooperative medical care, patients’ illness stories can be injected with more positive factors such as understanding, support, and hope, thus achieving a healing transformation. On the other hand, the medical personnel involved in the medical experience and the story co-constructed with their patients will also have an enriched experience of human nature and life and deepened their medical beliefs, thus nourishing their practical ability to care for and attend to their patients.

Although some comparisons could be made between conventional medicine (as well as modern technically enhanced biomedical approaches) and narrative medicine, they should not be viewed as an absolute dichotomy. As well summarized by Milota, van Thiel, and van Delden [12], “better attention to and appreciation of narratives in clinical settings can help doctors bridge gaps between their mediopathological knowledge and the experiential knowledge contained in their patients’ stories”, thus, the application of narrative medicine with narrative competence could be advantageous to meet the growing demand for patient-centered services and shared decision-making in a more diverse modern medical environment.

Health professionals who value patient-centered medical services, and take note of the psychosocial issues of patients, may prefer narrative medicine and consider this method meaningful and approachable. In other words, they can relate to this emerging model by sharing the commonalities between traditional medicine and narrative medicine, while drawing on or referring to their experience and expertise.

Regardless of their treatment orientation, narrative medicine-informed health practitioners may take advantage of the multi-dimensional narrative competence of this model, as presented in this article, in order to strengthen their awareness and readiness in different competence areas. Despite criticism, and even some tension, the ongoing dialogue between biomedical tradition and narrative medicine will enrich our understanding and reflections of patients, diseases, treatments, and human life, and thereafter, as medicine advances, we can anticipate more integrated and complementary medical services.

Compared with the narrative competence mentioned by Rita Charon, the findings of this study were more specific. For example, Charon’s narrative competence refers to the ability to acknowledge, absorb, interpret and be moved, but it is a major challenge how to translate these words to make them the content for the medical education. This study found that part of the connotation of the narrative construction subtheme generally includes Charon’s definition of narrative, but at the same time, it is richer and more layered in the connotation theme. In addition, Charon believes that a practitioner with narrative competence will show such characteristics as empathy, reflection, professionalism, and trustworthiness in clinical practice [1]. In this study, it was also found that these are important traits in medical relationships. However, this study found that responsive and balanced medical care that addresses to patient needs can complement the deficiency of the past literature.

For patients with chronic and major diseases, feeling ill means not only symptoms and duration but also a series of life changes that are full of stories of sadness and sporadic encouragement. Through illness story-telling and reading, medical students can learn more about the overall phenomenon of falling ill, perceive the patient’s subject experience and situation, and identify key psychosocial issues. Focusing on the narrative and reflective process of disease helps develop the ability of narrative listening and narrative understanding to construct a patient-centered perspective.

Narrative frames can be further used as an epistemology of narrative medicine education. With such ability, medical personnel can understand that to heal is to help patients to find peace and wellness while fighting for recovery from their illnesses. This understanding and frame require medical students and medical professionals to be aware of how patients go through and cope with illness. In order to achieve peace and wellness, interdisciplinary collaboration that attends to patients’ different needs is essential from a narrative perspective. An in-depth understanding and appreciation for the function of different medical personnel and active dialogues among them will facilitate such collaboration, which should be cultivated in the early stage of medical education [29].

Often, treatment requires alliances and cooperation among patients and physicians as well as other medical personnel. Building sufficient understanding and trust among all players is relevant to the course and outcome of the treatment. Medical education programs that value medical relationships and provide practical training should develop students’ relationship competence to demonstrate abilities for empathy, communication, affiliation, and inter-subjectivity in the treatment context. More detailed case observations and reflections and an emphasis on medical interaction will not only help students internalize relationship competence but also enhance their confidence to work with different patients and colleagues [30]. It will be useful for medical teachers to provide feedback to reinforce such learning processes.

Healthcare teamwork and interprofessional collaborations involve a great deal of quality communication. Likewise, as communication is the basis of the doctor-patient relationship and treatment, the importance of communication has been widely recognized by medical education and medical services. Incorporating narrative competence into the medical school curriculum could make a unique contribution to facilitating communication among patients, doctors, and medical personnel. By combining storytelling (stories of patients and students), diary writing, and reflection activities, medical teachers can help students in an experiential manner to identify, absorb, and interpret the illness and treatment stories constructed by patients and medical staff, and be moved [24, 31]. This process will help students develop empathy, different perspectives, and mutual respect, which will ultimately enhance medical communications and relationships. The improvement of the overall narrative competence, especially in the areas of narrative construction (i.e., narrative listening, narrative understanding, etc.) and medical relations (i.e., affiliation, intersubjectivity, etc.), will empower medical staff to communicate and interact more effectively with patients and colleagues.

Narrative medicine-reinforced medical practice assists medical work in meeting the growing trend of placing emphasis on medical ethics and patient autonomy. Medical professionals with narrative care are committed to responsive care through the efforts of balanced acts and face ambiguity and uncertainty in the medical process through reflection. According to the results of this study, the medical reflection and responsive care found in the narrative perspective emphasize the patient’s subjectivity, participation, related connections, the value and adoption of their opinions, and assist in balancing uncertainty and creating individualized medical care in a rapidly changing medical environment. In fact, this responds to practice-based learning and improvement (PBLI), which is one of the six core competencies highlighted in the current medical education field [32, 33] and the spirit of participatory medicine [34]. The establishment of the narrative competence will lead medical members to reflect on the past from the perspective of their patients, implement self-learning, and find continuous motivation for patients to make continuous progress.

## Conclusion

Narrative medicine can enhance the professionalism of medical personnel, and finding the kind of narrative competence medical personnel need to have in order to clinically approach their patients’ illness experience was the main purpose of this study. Using the method of qualitative inquiry, this study conceptualized the four dimensions/themes of narrative horizon, narrative construction, medical relationship, and medical care, as well as 12 subthemes of narrative compatence. Among these subthemes, the patient-centered frame was found to be the core of the epistemology of narrative medicine education. On this basis, medical personnel should have the ability to listen and understand, and at the same time have the narrative thinking and representation ability needed to approach the patient and construct a patient-based illness story. In clinical practice, medical personnel must be able to establish a relationship of mutual communication with the patient so that they can reflect from time to time and construct medical care that responds to the patient’s needs.

Previous related literature has found that narrative medicine education programs generally have a positive impact [12], but there is little research on the measuring the outcome of medical personnel’s narrative competence. This study initially generalized the conceptual framework of medical personnel’s narrative competence, which can be applied in clinical and medical education, especially in the three-step reading-reflection-responding process of narrative medicine education and training, to further evaluate the effect of narrative medicine training programs on improving medical personnel’s narrative competence.

## Supplementary Information

**Additional file 1.** Interview Protocol (Interview Guide)

## Data Availability

The datasets (interview transcripts) generated and/or analyzed during the current study are not publicly available due to concerns regarding compromise of participant confidentiality.

## Abbreviations

NC: Narrative competence
NM: Narrative medicine
