# Supplementary material for: A qualitative study of clinical narrative competence of medical personnel
Source: BMC Med Educ. 2020 Nov 10;20:415. doi: 10.1186/s12909-020-02336-6 (PMC7653871; doi:10.1186/s12909-020-02336-6)
Supplement: Supplementary file 1 — Additional file 1. Interview Protocol (Interview Guide) [file 12909_2020_2336_MOESM1_ESM.docx]

Interview Protocol (Interview Guide)

1. Please introduce your medical (or professional) background, training and practices.
2. Under what circumstances or occasions were you exposed to narrative medicine (NM)? What are your relevant training, education, and research experiences regarding NM?
3. What is your viewpoint and concept of NM? Are there any similarities or differences between the current mainstream logic and scientific-based medical knowledge?
4. What is your understanding and opinion about the concept of narrative competence (NC) of medical personnel?

4-1 What is your understanding and opinion about Rita Charon's definition of NM and NC? (We provided the definition of NM and NC by Rita Charon before the interviews.)

4-2 What are your understandings and opinions on the definition of NM and NC by other scholars?

1. How do you incorporate the concept of narrative medicine into your teaching activity?
2. How do you incorporate the concept of NM into your practice?
3. What do you expect the differences in the students’ clinical practice will be after receiving narrative medicine training?
4. What is the difference in your care for patients after incorporating narrative medicine in your clinical practice?
5. What will be the obstacles (questioning, challenges, and resistance) to promoting NM in medical education and clinical practice?
6. What narrative competencies are necessary for medical students in your medical education experience?
7. What narrative abilities are needed for medical personnel in your clinical experience?
8. Which narrative competencies are necessary for medical students in your medical education experience?
9. Which narrative competencies are needed for medical personnel in your clinical experience?
10. What narrative competencies do you think can be applied, and how can they be applied in clinical care?
11. Others
